# Retreat and extinction of the Late Pleistocene cave bear (***Ursus spelaeus*** sensu lato)

**DOI:** 10.1007/s00114-016-1414-8

**Published:** 2016-10-11

**Authors:** Mateusz Baca, Danijela Popović, Krzysztof Stefaniak, Adrian Marciszak, Mikołaj Urbanowski, Adam Nadachowski, Paweł Mackiewicz

**Affiliations:** 1 Center for Precolumbian Studies, University of Warsaw, Krakowskie Przedmieście 26/28, 00-927 Warszawa, Poland; 2 Institute of Genetics and Biotechnology, University of Warsaw, Pawińskiego 5A, 02-106 Warszawa, Poland; 3 Centre of New Technologies, University of Warsaw, S. Banacha 2c, 02-097 Warszawa, Poland; 4 Department of Paleozoology, Institute of Environmental Biology, University of Wrocław, Sienkiewicza 21, 50-335 Wrocław, Poland; 5 Department of Archaeology, Institute of History and International Relations, Szczecin University, Krakowska 71-79, 71-017 Szczecin, Poland; 6 Institute of Systematics and Evolution of Animals, Polish Academy of Sciences, Sławkowska 17, 31-016 Kraków, Poland; 7 Department of Genomics, Faculty of Biotechnology, University of Wrocław, Joliot-Curie 14a, 50-383 Wrocław, Poland

**Keywords:** Ancient DNA, Cave bear, Extinction, Last glacial maximum, Megafauna, Refugium

## Abstract

The cave bear (*Ursus spelaeus* sensu lato) is a typical representative of Pleistocene megafauna which became extinct at the end of the Last Glacial. Detailed knowledge of cave bear extinction could explain this spectacular ecological transformation. The paper provides a report on the youngest remains of the cave bear dated to 20,930 ± 140 ^14^C years before present (BP). Ancient DNA analyses proved its affiliation to the *Ursus ingressus* haplotype. Using this record and 205 other dates, we determined, following eight approaches, the extinction time of this mammal at 26,100–24,300 cal. years BP. The time is only slightly earlier, i.e. 27,000–26,100 cal. years BP, when young dates without associated collagen data are excluded. The demise of cave bear falls within the coldest phase of the last glacial period, Greenland Stadial 3. This finding and the significant decrease in the cave bear records with cooling indicate that the drastic climatic changes were responsible for its extinction. Climate deterioration lowered vegetation productivity, on which the cave bear strongly depended as a strict herbivore. The distribution of the last cave bear records in Europe suggests that this animal was vanishing by fragmentation into subpopulations occupying small habitats. One of them was the Kraków-Częstochowa Upland in Poland, where we discovered the latest record of the cave bear and also two other, younger than 25,000 ^14^C years BP. The relatively long survival of this bear in karst regions may result from suitable microclimate and continuous access to water provided by deep aquifers, indicating a refugial role of such regions in the Pleistocene for many species.

## Introduction

The extinction of large-bodied mammals (called megafauna) is one of the most characteristic and inherent features of the Late Pleistocene. The disappearance began 50,000 years ago and affected a substantial number of mammalian genera, e.g. 36 % of them in Eurasia, 72 % in North America and 83 % in South America (Barnosky et al. 2004). Both the climate and environment changes, as well as human influence, are believed to be the main causes of this extinction (Barnosky et al. 2004; Cooper et al. 2015; Koch and Barnosky 2006; Lorenzen et al. 2011; Stuart 2015). The climate shift was sufficient to explain the fauna transformation in some cases, but in others, a combination of climatic and anthropogenic effects was most probably responsible for this phenomenon (Cooper et al. 2015; Lorenzen et al. 2011).

A typical representative of megafauna is the cave bear (*Ursus spelaeus* sensu lato), which was one of the most widespread mammals in Eurasia in the Late Pleistocene. It evolved from Middle Pleistocene *Ursus deningeri* and developed into several forms which can be distinguished at morphological and genetic levels. Two main European forms in the species rank, which diverged probably between 414,000 and 173,000 years ago, were identified as *Ursus ingressus*, which inhabited south-eastern and central Europe as well as the Ural (Baca et al. 2014; Rabeder et al. 2004b), and *U. spelaeus*, which lived mainly in western Europe, although its remains were found also in the Altai (Knapp et al. 2009; Rabeder et al. 2004b). According to the rules of the International Code of Zoological Nomenclature, *U. ingressus* should, however, be called *Ursus kanivetz*, because under the latter name a bear from Medvezhiya Cave in the Ural was first described by Vereshchagin (1973) (see also Baryshnikov and Puzachenko (2011)). Further studies of ancient DNA showed that the haplotype from Medvezhiya Cave is clustered with others from Europe, described as *U. ingressus* (Baca et al. 2012; Knapp et al. 2009). Additionally, two small cave bear forms that had preserved some primitive traits were distinguished as subspecies of *U. spelaeus*: *U. spelaeus eremus* and *U. spelaeus ladinicus* (Rabeder and Hofreiter 2004; Rabeder et al. 2004a)*.* Their distribution was confined to the high alpine caves in Austria and Italy. Recently, another major group of large cave bears from the Caucasus and the Yana River region in eastern Siberia was discovered (Baryshnikov 1998; Knapp et al. 2009). Initially, they were named *Ursus deningeri kudarensis*, but recent genetic studies suggest that they should be considered a third species, *U* *rsus kudarensis* (Stiller et al. 2014).

By the end of the Pleistocene, all these cave bear forms were extinct and the causes and timing of this process have been debated over the recent years. Direct radiocarbon dating indicates that the last cave bears became extinct prior to the Last Glacial Maximum (LGM) and disappeared from fossil record quite simultaneously in different parts of Europe about 24,000 ^14^C years before present (BP) (about 28,000 cal. years BP) (Bocherens et al. 2014; Hofreiter et al. 2002; Martini et al. 2014; Pacher and Stuart 2009; Sabol et al. 2014; Wojtal et al. 2015). Paleogenetic analyses showed, nonetheless, that the demise of cave bears started ca. 50,000 radiocarbon years BP (Stiller et al. 2010), thus about 25,000 years before their final extinction. It has been argued that apart from the changing climate (Pacher and Stuart 2009; Stuart and Lister 2007), several other factors contributed to the decline of cave bears. There is compelling evidence for human hunting of cave bears (Münzel et al. 2011; Wojtal et al. 2015), as well as their competition for caves as a shelter (Grayson and Delpech 2003). Possibly, also large carnivores like cave lion (*Panthera spelaea*) and cave hyena (*Crocuta crocuta spelaea*) hunted cave bears while these were hibernating (Bocherens et al. 2011a; Diedrich 2014).

The paper reports on, so far, the youngest remains of the cave bear from the Stajnia Cave located in the Częstochowa Upland, Poland. In this region were also found other quite young fossils of this bear in two caves, Komarowa and Deszczowa (Nadachowski et al. 2009; Wojtal 2007; Wojtal et al. 2015). Genetic analyses confirmed beyond doubt the affiliation of this specimen to the cave bear, whereas the direct radiocarbon dating provided the evidence for the survival of this species into the Greenland stadial GS-3. Using this new dating and more than 200 published dates, we estimated the time of cave bear extinction and discussed potential factors of its disappearance and survival in karst regions.

## Materials and methods

### Excavation site and specimen description

**Fig. 1:**
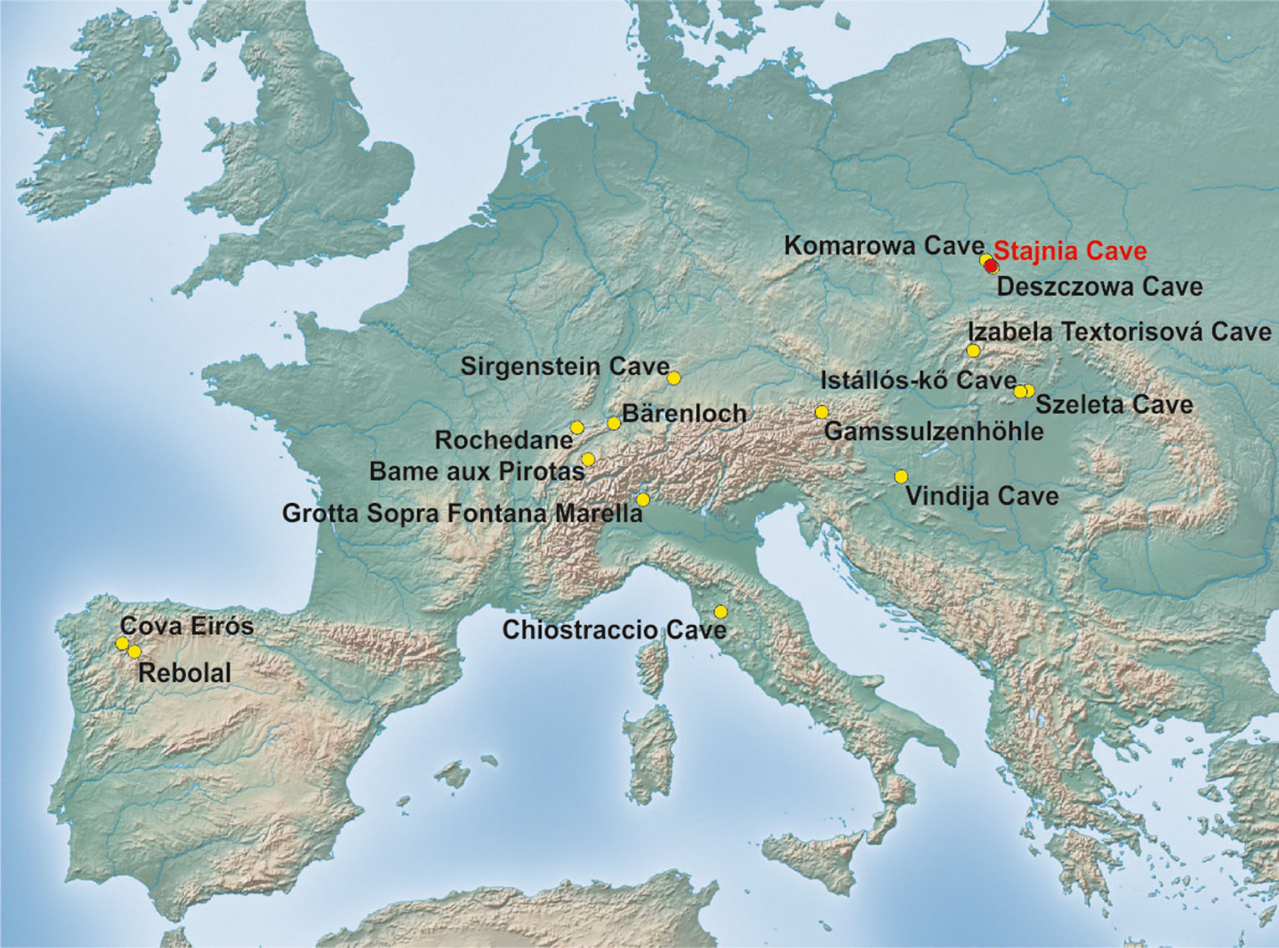
Localization of cave bears (*Ursus spelaeus* sensu lato) remains younger than 26,000 ^14^C years BP in Europe including the latest record confirmed genetically from the Stajnia Cave

**Fig. 2:**
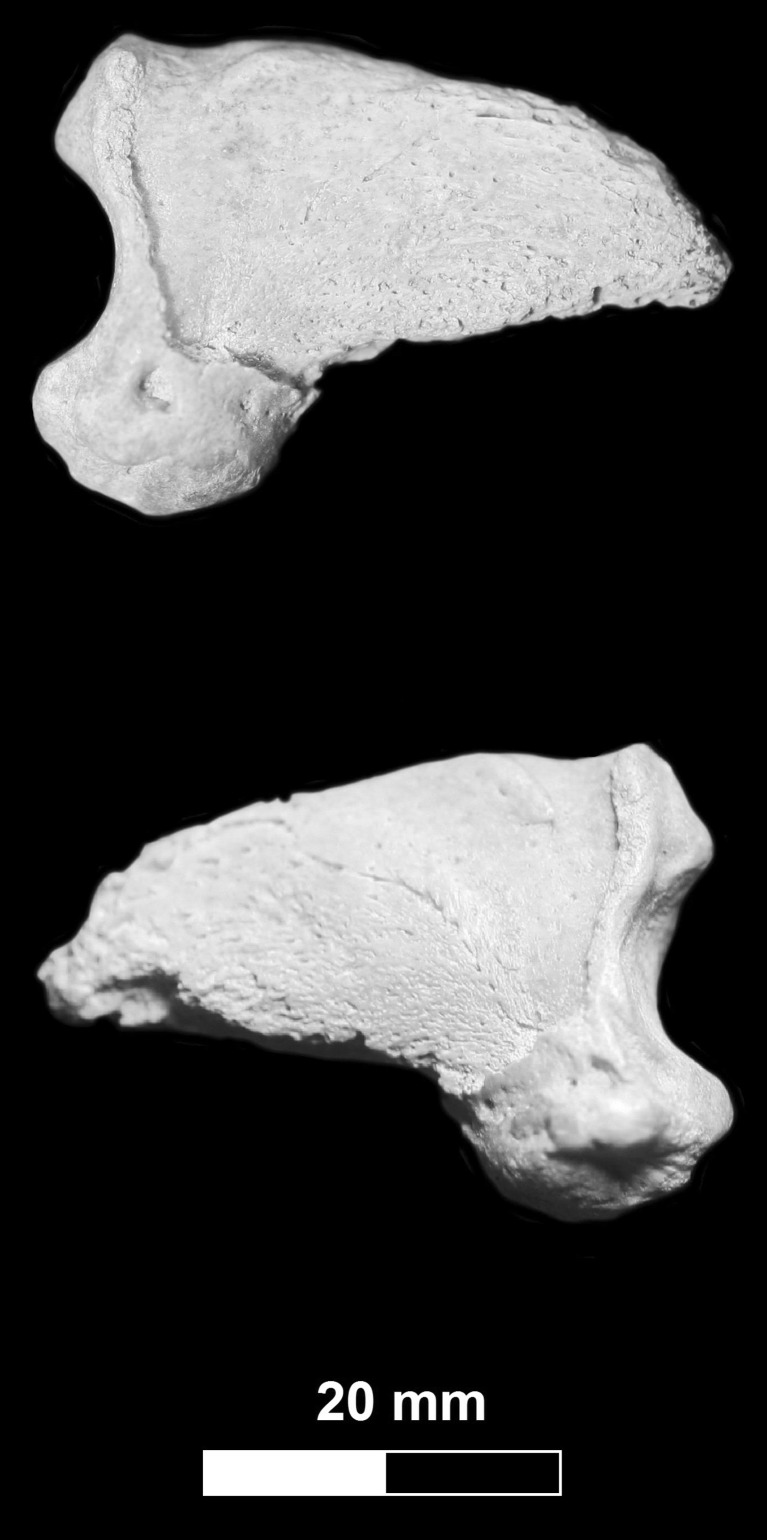
The third phalanx (JST4) excavated in the Stajnia Cave and confirmed genetically as belonging to the cave bear *Ursus ingressus*

The specimen of the cave bear (JST4), the third phalanx, was excavated in Stajnia Cave located in the Kraków-Częstochowa Upland in Poland (50° 36′ 58″ N, 19° 29′ 04″ E, Fig. 1). The specimen under study shows a morphometry typical of speleoid bear forms (Fig. 2). It had the greatest length = 39.3 mm and the proximal height = 27.1 mm. The measurements better correspond to the cave than brown bear. Mean and standard deviation of these parameters are respectively 38.3 ± 3.7 and 25.9 ± 2.3 mm for *U. spelaeus* from Buse di Bernardo (Italy) (Santi et al. 2011) and 36.6 ± 5.5 and 25.1 ± 3.7 mm for *U. ingressus* from Gamssulzenhöhle (Austria) (Alscher 2013), whereas subfossil *Ursus arctos* from Austria and France is characterized by smaller dimensions, i.e. 33.8 ± 5.8 and 20.9 ± 6.0 mm, respectively (Alscher 2013). Therefore, the measurements for the specimen JST4 are closer to the mean values of the cave bear and are much higher than those for the brown bear. Moreover, the phalanx is more massive and not as slender as in the brown bear. It has also a well-developed articular surface with subcircular contour (Bonifay 1966; Torres Pérez-Hidalgo 1989).

The Stajnia Cave is famous for the discovery of the first remains of Neanderthals in Poland (Nowaczewska et al. 2013; Urbanowski et al. 2010). Six stratigraphical complexes named from A to G were distinguished in this cave, spanning the period of more than 100,000 years (Żarski et al. 2017). The youngest complexes A and B represent MIS 1 sediments. The complex C consists of several layers, marked from the top as C6, C7 and C18 corresponding to MIS 2 as well as C19 from the younger MIS 3. The deposits consist of poorly sorted sandy loams containing sharp-edged limestone rubble, dated to the LGM. The cave bear specimen under study was found at the bottom of layer C18 deposited during the Leszno (Brandenburg) Phase. The complex D of about 60 cm average thickness includes three units D1, D2 and D3, dated to the older part of MIS 3. They yielded the remains of *Homo neanderthalensis* and very rich Late Middle Palaeolithic flint artefacts. The archaeologically sterile complex E is most probably of MIS 4 age, whereas the oldest complexes F and G are dated to MIS 5. Remains of the Late Pleistocene cave bear (*U. spelaeus* sensu lato) were found in large numbers throughout the whole profile from layers G to C6. A tooth of cave bear from layer D1 was dated to >49,000 years BP. In total, 323 teeth and bones of the bear were found in this cave.

### Laboratory analysis of specimen

The bone was cut into two parts and one of them was submitted to radiocarbon dating in Poznań Radiocarbon Laboratory, whereas the other one was used for genetic analyses. DNA extraction was performed in a laboratory dedicated to ancient DNA analyses observing strict contamination precautions. Samples were thoroughly cleaned with sterile toothbrush and bleach, rinsed with ddH_2_O and pulverized in cryogenic mill (Spex). A portion of the obtained bone powder was used for DNA extraction following the established protocol (Baca et al. 2012). A 309-bp-long fragment of mitochondrial DNA control region was amplified in singleplex PCR reactions with five different primer pairs (Baca et al. 2012). PCR products were pooled and converted into Illumina sequencing library following the protocol proposed by Meyer and Kircher (2010). Two uniquely indexed libraries based on independently amplified PCR products were produced, pooled with other libraries and sequenced on MiSeq platform. Adaptor and quality trimming were performed with Adapter Removal (Lindgreen 2012). PCR primer sequences were trimmed in Mothur (Schloss et al. 2009). The readings were assembled in SeqMan Pro (DNASTAR). Consensus sequences from two replicates were called according to the guidelines proposed by Stiller et al. (2009). To further confirm AMS date obtained in Poznań Radiocarbon Laboratory, the remaining bone powder (ca. 300 mg) was sent to GADAM Centre in Gliwice, Poland, for independent dating.

### Analysis of DNA sequence

To verify taxonomic and phylogenetic position of the analysed specimen, we compared it with all 141 sequences of cave bears (*U. spealeus*, *U. ingressus*, *U. rossicus* and *U. kudarensis*) available in GenBank as well as 490 sequences of brown bear (*U. arctos*). The number of base differences per site (*p* distance) between the sequence under study and the others was calculated in MEGA6 (Tamura et al. 2013). The median-joining network (Bandelt et al. 1999) was constructed using the Network 4.6.1 software (fluxus-engineering.com). The MP algorithm was used to resolve reticulations in the final network (Polzin and Daneschmand 2003).

Phylogenetic trees were created by Bayesian method in MrBayes 3.2.3 (Ronquist et al. 2012) and maximum likelihood in morePhyML 1.14 (Criscuolo 2011) based on PhyML 3.0 (Guindon et al. 2010). In the MrBayes analyses, we adopted a mixed model to sample appropriate substitution models across the larger space in the Bayesian MCMC analysis itself, avoiding the need for a priori model testing (Huelsenbeck et al. 2004). Additionally, we applied gamma-distributed rate variation across sites with five discrete rate categories as suggested by jModeltest 2.1 based on Bayesian Information Criterion (BIC) and decision theory (DT) criterion (Darriba et al. 2012; Guindon et al. 2010). Two independent runs starting from random trees, each using eight Markov chains, were applied. The trees were sampled every 100 generations for 20,000,000 generations. In the final analysis, we selected trees from the last 5,500,000 generations that reached the stationary phase and convergence (i.e. the standard deviation of split frequencies stabilized and was lower than the proposed threshold of 0.01). The tree inferred in morePhyML was based on the best-fit substitution model TPM2uf+Γ found in jModeltest 2.1 among 1624 candidate models according to BIC and DT criteria. The best heuristic search algorithm, nearest neighbour interchanges (NNI) and subtree pruning and regrafting (SPR), in morePhyML was applied. The non-parametric bootstrap analysis in PhyML was carried out applying 1000 replicates and assuming the approximate likelihood ratio test (aLRT) based on a Shimodaira-Hasegawa-like procedure in morePhyML.

### Estimation of extinction time

To determine the extinction time of the cave bear, we collected 207 dates of its remains. The dates were carefully selected from the set of 513 dates reported in various references, including an excellent and comprehensive review of dating the cave bear remains done by Pacher and Stuart (2009). We discarded the dates based on molecular, uranium series, uranium/thorium, stratigraphy context and strata dating, dates of *U. kudarensis* remains, without dating error, infinitive dates and dates out of range 47,500 ± 3000 BP after calibration, as well as the dates of remains with unclear affiliation to the cave bear. All the dates were calibrated to the years BP in OxCal v4.2.4 (Bronk Ramsey et al. 2013) using intCal13 atmospheric curve (Reimer et al. 2013). In the assessment of the extinction time, calibrated mean values and standard deviations were used.

The best-fitted distribution to the set of dates was selected adopting the Akaike information criterion (AIC) and Schwarz Bayesian criterion (BIC) based on the maximum likelihood method applying fitdist from library fitdistrplus in R package (R_Core_Team 2015). Besides R package, statistical analyses were also performed in Statistica (StatSoft_Inc. 2011). To define the extinction time, we performed a procedure based on five methods devised by Strauss and Sadler (1989), Solow (1993), Roberts and Solow (2003), Solow and Roberts (2003) and McInerny et al. (2006) and implemented by Rivadeneira et al. (2009). In addition, we applied the newly developed inverse-weighted McInerney Gaussian-resampled (GRIWM) (Bradshaw et al. 2012) and bootstrap-resampled (BRIWM) methods (Saltré et al. 2015). In the last two approaches, we assumed 10,000 iterations and *α* level 0.05.

## Results

### Dating sample from Stajnia Cave

Direct radiocarbon dating the JST4 sample in Poznań Radiocarbon Laboratory yielded an unexpected young ^14^C date 20,930 ± 140 years BP. Collagen yield was low (ca. 2 mg after ultrafiltration, 0.3 % of initial sample’s weight) but the computed C/N ratio (3.6) was close to the upper limit of the accepted range: 2.9–3.6 (DeNiro 1985; van Klinken 1999). To verify this date, the remaining bone powder was dated in Gliwice Absolute Dating Methods Centre for independent replication. The GADAM extraction procedure yielded more than 3.5 mg of collagen from the processed bone sample and resulted in the ^14^C date 21,900 ± 90 years BP, still quite young. The C/N ratio was estimated at a similar level. After calibration, the second date obtained for the JST4 sample is older (26,114 cal. years BP) than the first one (25,251 cal. years BP) and their 95 % probability intervals (25,648–24,807 and 26,360–25,905 cal. years BP, respectively) do not overlap (Table 1). The dates are, however, similar because the extreme values of their intervals differ in only 257 years. Therefore, this double check suggested a high degree of reliability of the dating.

**Table 1:** Direct radiocarbon dates of cave bear remains younger than 26,000 ^14^C years BP

Locality	Country	Specimen	Sample number	Unc. date years BP ± error	Calibrated date years BP			Haplotype	Source reference
					Mean ± SD	Median	95 % CI		
Stajnia Cave	Poland	3rd phalanx	Poz-61719	20,930 ± 140^a^	25,251 ± 205	25,267	25,648–24,807	*U. ingressus*	This paper
Grotta Sopra Fontana Marella	Italy	?	UZ-2512/ETH-5I98	21,810 ± 200^a^	26,082 ± 202	26,058	26,516–25,712	*–*	Perego et al. (2001)
Stajnia Cave	Poland	3rd phalanx	GdA-3894	21,900 ± 90^a^	26,114 ± 113	26,101	26,360–25,905	*U. ingressus*	This paper
Vindija Cave	Croatia	?	Beta-171313	22,020 ± 100^a^	26,235 ± 137	26,221	26,516–25,985	*U. ingressus*	Hofreiter et al. (2004b)
Szeleta Cave	Hungary	Bone	ISGS-A-0131	22,107 ± 130^a^	26,331 ± 175	26,317	26,681–26,006	*–*	Adams (2002)
Grotta Sopra Fontana Marella	Italy	?	UZ-2513/ETH-5199	22,310 ± 200^a^	26,600 ± 273	26,585	27,122–26,102	*–*	Perego et al. (2001)
Chiostraccio Cave	Italy	Long bone	Beta-285012	22,670 ± 130	26,990 ± 197	27,010	27,340–26,595	*–*	Martini et al. (2014)
Rebolal Cave	Spain	Adult jaw	Ua-24939	22,915 ± 445^a^	27,128 ± 429	27,161	27,886–26,202	*–*	Grandal-d’Anglade et al. (2006)
Vindija Cave	Croatia	?	Beta-156100	23,780 ± 120^a^	27,851 ± 120	27,841	28,103–27,624	*U. ingressus*	Hofreiter et al. (2004a)
Rochedane	France	3rd metatarsal	GrA-52632	23,900 ± 110	27,948 ± 134	27,934	28,231–27,701	*U. spelaeus*	Bocherens et al. (2014)
Chiostraccio Cave	Italy	Phalanx	Beta-340969	23,930 ± 100	27,969 ± 132	27,956	28,245–27,725	*–*	Martini et al. (2014)
Cova Eirós	Spain	Humerus	Ua-4298	24,090 ± 440	28,237 ± 405	28,205	29,070–27,475	*–*	Grandal-d’Anglade and Vidal Romani (1997)
Bame aux Pirotas	Switzerland	Metapodial frag.	ETH-16879	24,170 ± 230	28,223 ± 237	28,216	28,679–27,783	*–*	Morel and Schifferdecker (1997)
Bärenloch	Austria	Radius	Ua-24796	24,175 ± 365	28,271 ± 343	28,249	28,956–27,634	*–*	Bochud et al. (2007)
Komarowa Cave	Poland	Skull	Poz-339	24,550 ± 220	28,582 ± 248	28,587	29,075–28,050	*–*	Wojtal et al. (2015)
Deszczowa Cave	Poland	Mandible frag.	Poz-28284	24,580 ± 200	28,615 ± 227	28,619	29,069–28,129	*U. ingressus*	Wojtal et al. (2015)
Izabela Textorisová Cave	Slovakia	Metacarpal IV	VERA-5679	24,640 ± 170	28,680 ± 193	28,680	29,076–28,280	*–*	Sabol et al. (2014)
Istállós-kő Cave	Hungary	Caudal vertebra	OxA-16640	24,950 ± 140	28,997 ± 183	28,983	29,379–28,660	*–*	Davies and Hedges (2008–2009)
Gamssulzenhöhle	Austria	?	VRI-1159	25,090 ± 640	29,310 ± 699	29,266	30,696–28,002	*–*	Fiebig and Pacher (2006)
Istállós-kő Cave	Hungary	Metacarpal	OxA-16639	25,500 ± 210	29,659 ± 314	29,633	30,305–29,060	*–*	Davies and Hedges (2008–2009)
Sirgenstein Cave	Germany	m1	OxA-12013	25,560 ± 130	29,707 ± 224	29,681	30,199–29,310	*U. ingressus*	Hofreiter et al. (2007)
Cova Eirós	Spain	Rib	Ua-38460	25,592 ± 602	29,781 ± 625	29,784	30,959–28,622	*–*	Pérez-Rama et al. (2011)
Gamssulzenhöhle	Austria	?	Hv 16893	25,965 ± 780	30,093 ± 765	30,101	31,480–28,537	*–*	Withalm (2001)

The haplotype assignment was based on DNA studies

*CI* confidence interval

^a^Dates which were excluded in the conservative estimation of extinction time

The dates are in agreement with the results of multidisciplinary geological, geochemical, palaeobotanical, palaeontological and isotopic analyses of the Stajnia Cave (Nowaczewska et al. 2013; Urbanowski et al. 2010; Żarski et al. 2017). They seem to indicate that the layer C18, in which the specimen under study was found, was accumulated in cold climate conditions and corresponds to the Leszno (Brandenburg) Phase dated to 20.1–23.7 (±1.1 to 2.4) ka (Marks 2012; Marks et al. 2015).

### Genetic verification of Stajnia sample

**Fig. 3:**
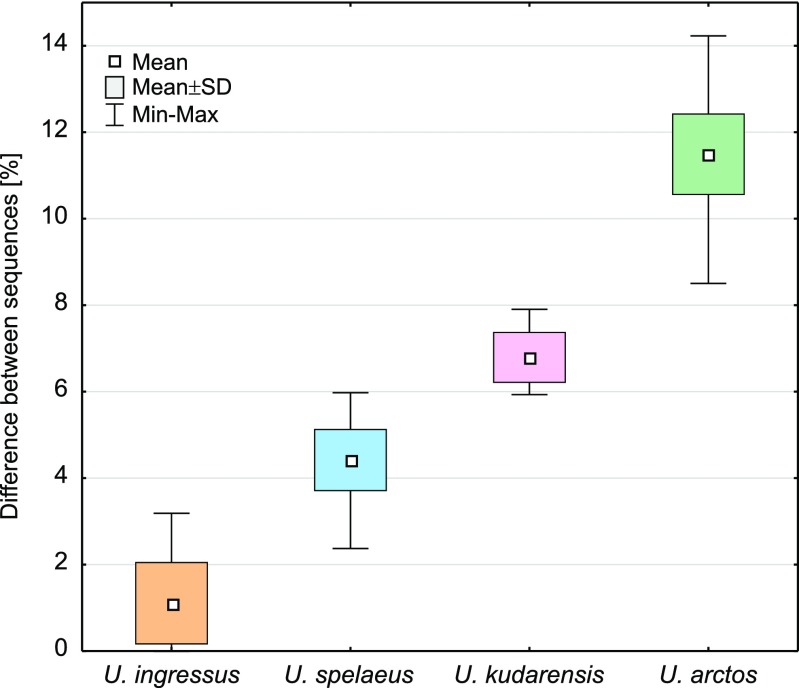
Differences between the sequence from the Stajnia Cave and other classified cave bears (*Ursus kudarensis*, *ingressus*, *spelaeus*) and the brown bear (*Ursus arctos*). The results clearly indicate a close similarity of the Stajnia sample to *Ursus ingressus*

The young dates could suggest that the sample belongs to the brown bear because it is assumed that cave bear disappeared about 24,000 ^14^C years BP (28,000 cal. years BP) (Bocherens et al. 2014; Hofreiter et al. 2002; Martini et al. 2014; Pacher and Stuart 2009; Sabol et al. 2014; Wojtal et al. 2015). Therefore, we verified its taxonomic assignment based on genetic studies. Analyses of sequence reads obtained from two independent libraries produced from the JST4 sample (11,340 and 10,208 reads mapped to the reference sequence, respectively) resulted in identical 309-bp-long consensus sequences. Having removed a poli-T/C stretching from the middle of the sequence and having trimmed 3′ end to fit the other available cave bear sequences, the 254-bp-long fragment was used for subsequent analyses. Comparisons of this sequence with all the available 631 sequences from cave and brown bears clearly indicated its affiliation to the cave bear classified to *U. ingressus* haplotype (Fig. 3).

**Fig. 4:**
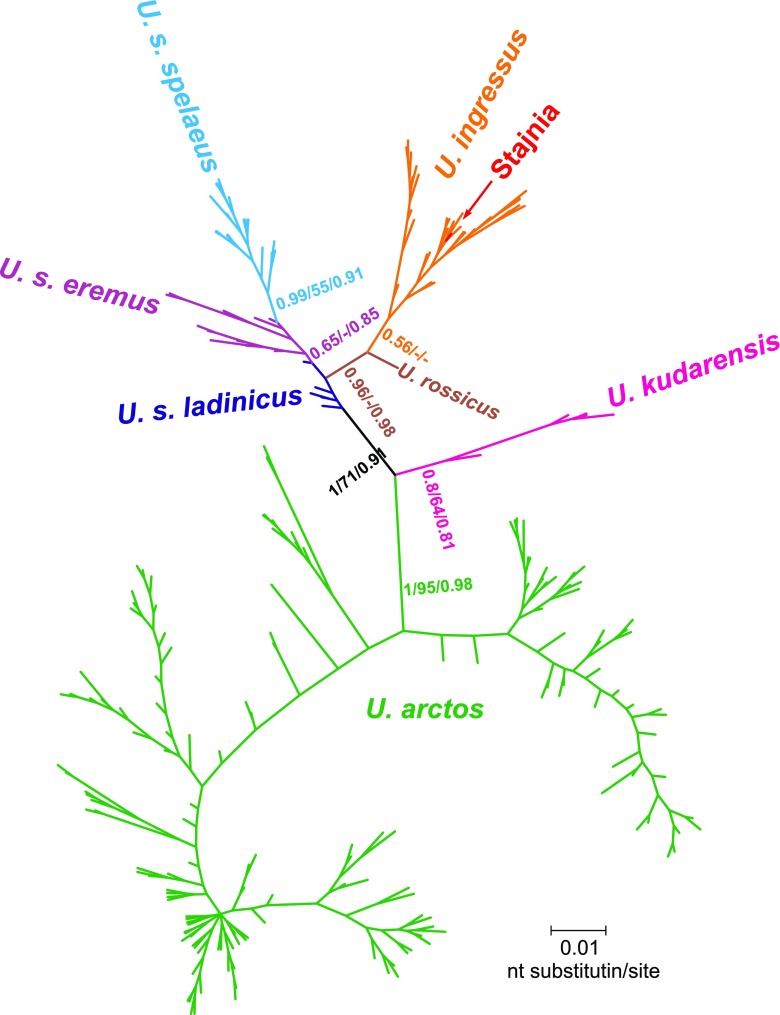
MrBayes phylogenetic tree for cave bears (*Ursus kudarensis*, *ingressus*, *rossicus*, *spelaeus*) and the brown bear (*Ursus arctos*) mtDNA control region. The sequence from the Stajnia sample indicated by an *arrow* groups significantly within *U. ingressus* clade. *Values at nodes* correspond in the order to: posterior probabilities estimated in MrBayes, support values calculated in morePhyML based on a Shimodaira-Hasegawa-like procedure and bootstrap values obtained in PhyML. Values for probabilities and bootstrap percentages lower than 0.50 and 50 %, respectively, were omitted

The sequence was identical with eight sequences isolated from the cave bear specimens excavated in Gamssulzen Cave, Austria (FM177760.1), Herdengel, Austria (FN663158.1), Nixloch, Austria (AJ300172.1, FN390842.1), Peştera cu Oase, Romania (EU289394.1) and Potocka Zijalka, Slovenia (AJ300173.1, FN390843.1, FN390844.1). The maximum percentage of differences with *U. ingressus* sequences was 3.2 % and averaged 1.1 %, whereas other bears differed from the Stajnia sequence by an average of 4.4 % (*U. spelaeus*), 6.8 % (*U. kudarensis*) and 11.5 % (*U. arctos*). The smallest difference from brown bear sequences was 8.5 %. It seems to suggest that it is improbable that the JST4 sample belongs to the brown bear (represented by almost 500 sequences) as it was suspected following the young radiocarbon date. In agreement with that, the Stajnia sequence grouped significantly with other *U. ingressus* sequences in phylogenetic trees (Fig. 4).

**Fig. 5:**
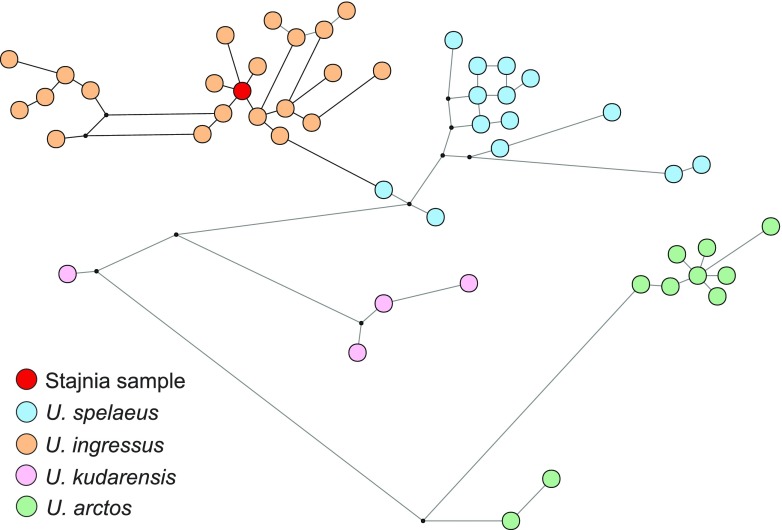
Haplotype network of cave bears (*Ursus kudarensis*, *ingressus*, *spelaeus*) and the brown bear (*Ursus arctos*). The haplotype from the Stajnia Cave occupies the central position within *U. ingressus* haplotypes

The tree confirmed separation of the cave bear into three main clades: *U. kudarensis*, *U. spelaeus* and *U. ingressus*. Interestingly, a *U. rossicus* sequence showed a closer affiliation to *U. ingressus*, whereas most of the sequences assigned to *U. spelaeus ladinicus* were located at the base of other modern cave bears, with the exclusion of *U. kudarensis*. This is indicative of *U. s. ladinicus* representing an early diverged lineage of cave bears related to ancestors of the modern cave bears. Median joining haplotype network placed the JST4 sample within the *U. ingressus* group, too (Fig. 5). Its haplotype occupies the central place in respect to others, which agrees with its quite wide distribution in Europe (Austria, Romania and Slovenia). It would arguably imply that the Stajnia sample, with its young radiocarbon age, is a remnant from the common haplotype which survived to the north of the Carpathian Mountains.

### Other young dates of the cave bear

**Fig. 6:**
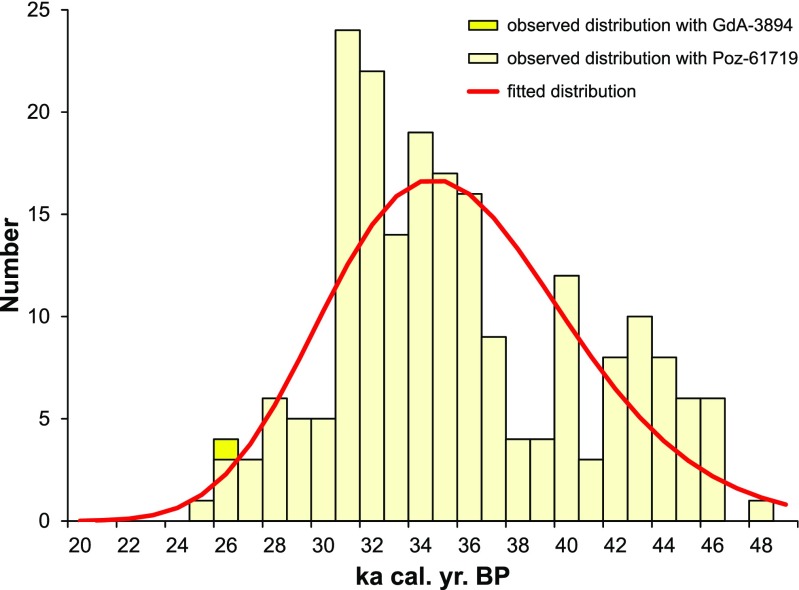
Distribution of the cave bear (*Ursus spelaeus* sensu lato) dates (*yellow bars*) with fitted density functions of lognormal distribution (*red line*). The function was averaged for two distributions with one of the dates for the Stajnia sample (Poz-61719 or GdA-3894)

In Table 1, we compared the dates of the cave bear remains younger than 26,000 ^14^C years BP reported so far, and in Fig. 6, we presented the distribution of collected cave bear dates. At the top of the list, there is the date for Stajnia Cave specimen (Poz-61719) and the next one is the date of the remains from Grotta Sopra Fontana Marella in Italy, albeit it was not genetically confirmed. The second youngest sample from which a DNA was extracted comes from Vindija Cave. It is almost 1000 years older than the Poz-61719.

The dates younger than 20,000 years were reported by other authors (see Pacher and Stuart (2009) for the review). However, they concerned samples that were carbonated or contaminated, which could rejuvenate the dates. Other young samples came from mixed fossil sets, therefore can represent the brown bear or other mammals. The other young date of *U. ingressus* 13,230 years BP from Geißenklösterle was revised by the second dating to 24,210 years BP (Münzel and Athen 2009). This date should nevertheless be considered with caution, too, as the collagen content of this sample is not reliable (Münzel et al. 2011). Similarly, the date 17,385 years BP obtained for a cave bear specimen from Bärenloch was also criticized as contaminated by recent carbon and another dating resulted in >46,900 years BP (Blant et al. 2010). It demonstrates that the radiocarbon dates, especially young, should be verified and the taxonomic assignment of such specimens should be checked by ancient DNA analyses, e.g. a sample from Rebolal Cave in Spain dated to 13,785 years BP (Grandal-d’Anglade et al. 2006) and from Urals sites dated to 16,470 years BP (Kosintsev et al. 2003). So far, the specimen from Stajnia remains the youngest case of the cave bear, which was twice dated and genetically studied. Only six other samples younger than 26,000 ^14^C years BP were verified by DNA studies (Table 1).

### Estimation of the cave bear’s extinction time

We estimated the time of the cave bear’s disappearance processing 207 dates of its remains by means of seven methods (Table 2). The calculations were based on two data sets. Since we obtained two dates for the Stajnia sample, only one of them was included in a given calculation to avoid a bias of this sample.

**Table 2:** Estimated extinction time of cave bear for seven methods and three data sets

Method	With Stajnia Cave date:		Conservative approach
	Poz-61719	GdA-3894	
Strauss and Sadler (1989)	24,910	25,754	26,664
Solow (1993)	24,912	25,755	26,665
McInerny et al. (2006)	24,916	25,759	26,669
Solow and Roberts (2003)	9462	25,474	8788
Roberts and Solow (2003)	21,266	25,327	22,731
BRIWM (Saltré et al. 2015)	24,540 (24,412–25,839)	25,725 (25,414–25,871)	26,378 (26,276–28,004)
GRIWM (Bradshaw et al. 2012)	24,749 (24,291–25,234)	25,720 (25,263–26,081)	26,555 (26,117–27,007)

For the first five methods, the upper boundary of 95 % confidence interval is presented. For BRIWM and GRIWM methods, median and 95 % confidence interval (in parentheses) are shown

Differences in the determined extinction time between sets with two Stajnia dates were very small (Table 2). The inclusion of the older Stajnia date (GdA-3894) resulted in the extinction time being older by only ca. 1000 years than the calculations with the younger date (Poz-61719). Generally, almost all the methods gave very similar mean extinction times of about 25,800 to 24,500 cal. years BP. This range is also inclusive of the time (24,807 cal. years BP) at two standard deviations for Poz-61719 date. Only the method by Roberts and Solow (2003) and Solow and Roberts (2003) produced deviated results. These approaches, however, are known to be the most conservative and are susceptible to the type II errors, giving confidence limits too broad, with the range greater than the nominal value. Their results can even indicate that an already extinct species should still be considered as extant (Rivadeneira et al. 2009). In fact, the method by Roberts and Solow (2003) produced a slightly younger extinction time, ca. 21,300 cal. years BP, and the method by Solow and Roberts (2003) an even much younger time, ca. 9500 cal. years BP for the set with Poz-61719 date.

The most reliable seems the GRIWM approach because it is the only one providing model accuracy and no misclassification issues. This is achieved by its inherent down-weighting interval procedure and by taking into account the uncertainties in record dates (Saltré et al. 2015). The range of 95 % confidence intervals based on this is ca. 26,100–24,300 cal. years BP and contains other 10 calculations (Table 2). It is 2000 to 3500 years later than it was previously assumed (Bocherens et al. 2014; Hofreiter et al. 2002; Martini et al. 2014; Pacher and Stuart 2009; Sabol et al. 2014; Wojtal et al. 2015) and 1600 to 2600 years later than the recent estimations using GRIWM, too, but with smaller sets of dates (Cooper et al. 2015).

Not all young dates listed in Table 1 provided chemical data about the collagen, and some of them were criticized for exactly this reason (Bocherens et al. 2014). These dates may not necessarily be wrong, but they should be confirmed by additional studies. Therefore, we also carried out an assessment of the extinction time excluding all the dates younger than the first youngest date for which collagen was well examined (Table 1). We did not consider in these calculations the dates from the Stajnia Cave specimen, either. This conservative approach for most of the methods gave extinction times older by merely 650–1800 years (Table 2). Only the methods by Roberts and Solow (2003) and Solow and Roberts (2003) delivered younger extinction times. According to the GRIWM methods, the most probable extinction time is in the range of ca. 26,100–27,000 cal. years BP.

**Fig. 7:**
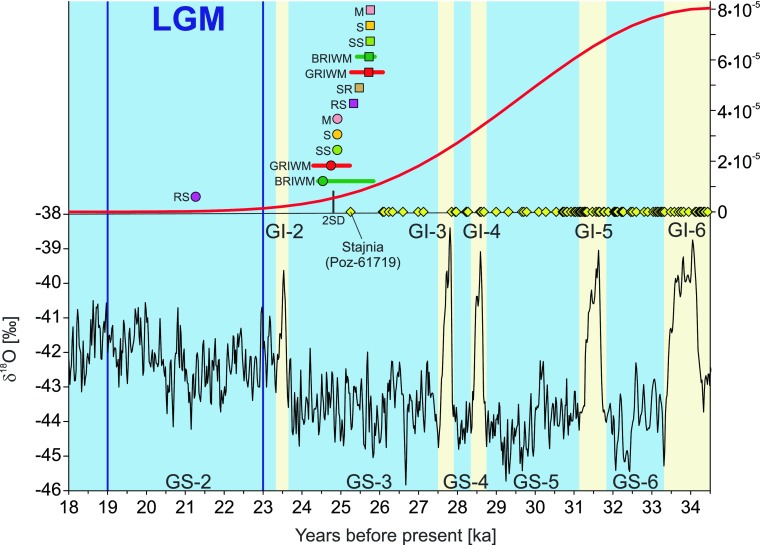
Distribution of late dates of the cave bear (*Ursus spelaeus* sensu lato) samples in the time scale (*yellow diamonds*). The youngest date of the Stajnia sample confirmed genetically was indicated (Poz-61719). The *red curve* in the upper part corresponds to the density function averaged for distributions with one of the dates for the Stajnia sample. *Symbols* above the curve indicate cave bear extinction times for two data sets, including the Stajnia Poz-61719 date (*circles*) or GdA-3894 date (*squares*), estimated by methods: *SS* Strauss and Sadler (1989), *S* Solow (1993), *M* McInerny et al. (2006), *SR* Solow and Roberts (2003), *RS* Roberts and Solow (2003), *BRIWM* Saltré et al. (2015) and *GRIWM* Bradshaw et al. (2012). *Horizontal bars* indicate 95 % confidence interval for BRIWM and GRIWM methods. Two standard deviations for the Poz-61719 date are also marked by a *short black vertical line* (2SD). The results were compared with the revised Greenland ice core δ^18^O curve (in *black*) developed by combining the Cariaco Basin (Hulu Cave) and Greenland ice core (GICC05) records (Cooper et al. 2015). Corresponding Greenland stadials (*GS*) and interstadials (*GI*) as well as strict definition of the Last Glacial Maximum (LGM) were marked. The results show that the cave bear became extinct in the middle of the cold stadial GS-3

Our calculated extinction times correspond to a δ^18^O decrease in the revised Greenland record developed by combining the Cariaco Basin (Hulu Cave) and Greenland ice core (GICC05) records (Cooper et al. 2015; Fig. 7). The estimated extinction time falls in the middle of Greenland Stadial 3 (GS-3) (or at the beginning in the conservative estimation) and matches also a significant decline in the density function describing the distribution of cave bear records (Fig. 7). The results indicate that the cave bear did not survive into the LGM, if we assume a rigorous definition of its duration from 23,000 to 19,000 cal. years BP (Waelbroeck et al. 2009). However, other researchers (see Hughes et al.(2013) for review) suggest that the global ice maximum started slightly earlier 26,000 (Peltier and Fairbanks 2006) or 26,500 cal. years BP (Clark et al. 2009). In this case, the species became extinct at the beginning of the LGM.

The estimated extinction time of cave bear corresponds to the end of the first megafaunal transition cluster, which began around interstadials 5 to 7 in northern Europe (Cooper et al. 2015). We also observed a gradual decrease in the number of reported dating records for the cave bear since that time (Fig. 7). Besides the cave bear also two other mammalian species disappeared at the end of this extinction event: the short-faced bear *Arctodus simus* in East Beringia and elephant *Palaeoloxodon naumanni* in Japan. The second cluster of extinctions occurred during the termination of the Pleistocene (ca. 14,000 to 11,000 cal. years BP) and concerned the species that survived the LGM. Our results indicate that the cave bear represents the pre-LGM megafaunal disappearance.

## Discussion

### Potential factors influencing cave bear extinction

A variety of causes of the cave bear extinction have so far been proposed (Kurten 1958; Münzel et al. 2011; Pacher and Stuart 2009; Stiller et al. 2010; Stuart and Lister 2007). Dramatic climate changes were most often reported. Climate deterioration began in Europe 33,000 cal. years BP and culminated in 24,000 cal. years BP, when Scandinavian Ice Sheet reached its maximum range (Clark et al. 2009; Marks 2012). Generally, the climate change at the end of the Last Glacial caused severe changes in plant communities in the whole of Europe and resulted in the considerable reduction of boreal forest and the spread of dry shrubland, steppes and tundra (Harrison and Prentice 2003; Ray and Adams 2001). Just before the estimated extinction time of the cave bear, between 28,000 and 27,000 cal. years BP, the climate in central Europe became extremely dry (Musil 2010). At around 27,000 cal. years BP, a significant cooling occurred, which resulted in the formation of cold continental steppe and forest steppe with tundra conditions. Further extreme cooling led to the development of continental climate in 25,000–23,000 cal. years BP and cold continental steppe or forest steppe with sites similar to tundra. Simultaneously, continuous permafrost developed in England, northern France, northern Belgium, the Netherlands, northern Germany and northern Poland with the lowest temperatures (Renssen and Vandenberghe 2003). Generally, the period 28,000–22,000 cal. years BP was characterized by a very cold and extremely arid climate in the whole of central and north-western Europe, resulting in the accumulation of loess and the formation of tundra gley soils. In the northernmost regions, climate resembled polar deserts. In this period, even southern Europe was dominated by xerophytic steppe, grassland and dry shrubland with only a small contribution of eurythermic conifers, as it is indicated by pollen records (Fletcher et al. 2010). As a result of the climate cooling, vegetation seasons shortened and the availability of high quality plant material decreased, which was crucial for cave bears’ survival because plants were the main source of its food.

Cave bears are generally considered herbivorous, following morphometric analyses of their skull and dentition (Grandal-d’Anglade and Lopez-Gonzalez 2004; Kurtén 1976; Mattson 1998; Rabeder et al. 2000; Van Heteren et al. 2009, 2014), limb proportions (Athen 2006) and bite forces in its jaws (Grandal-d’Anglade 2010). The herbivory of this bear is also supported by a relatively thick dental enamel cap (Mackiewicz et al. 2010) and several adaptations in enamel structure (Wiszniowska et al. 2010) to harder and more abrasive food items. Figueirido et al. (2009) came to the opposite conclusions based on morphometrical studies of bear skulls, but these studies were criticized by the lack of allometric correction and inclusion of all extant bear taxa, e.g. the early diverged giant panda (*Ailuropoda melanoleuca*) (Grandal-d’Anglade 2010; Van Heteren et al. 2009).

The vegetarian diet of the cave bear is also confirmed by many studies of stable isotopes (δ^13^C, δ^15^N) from bone and tooth collagen (Blant et al. 2010; Bocherens et al. 1994, 1997, 2006; Fernández-Mosquera et al. 2001; Horacek et al. 2012; Krajcarz et al. 2014; Nelson et al. 1998; Pacher et al. 2012; Vila Taboada et al. 1999). Having analysed isotope composition, no differences were found between still vegetarian habits of *U. spelaeus* and *U. ingressus* from Ach valley (Münzel et al. 2011). Similarly, coexisting *U. ingressus* and *U. spelaeus eremus* from Austrian caves Gamssulzen and Ramesch were also exclusively herbivorous, although they could consume different plant types and/or plants from different habitats (Bocherens et al. 2011b; Münzel et al. 2014). The most comprehensive isotopic studies of cave bear samples from 60,000–25,000 cal. years BP (MIS 3) showed neither taxonomic nor geographic pattern both in east-west and south-north directions (Krajcarz et al. 2016). The most distinct isotopic sets came only from high Alpine sites located over 1500 m above sea level, as well as two Romanian sites Peştera cu Oase and Urşilor. Some isotopic gradient with altitude was also observed. The studies indicated that the cave bear was homogeneous in the global scale according to its vegetarian diet and was characterized by low ecological flexibility.

On the other hand, some isotope analyses were indicative of an omnivorous diet for cave bears (Hildebrand et al. 1996; Richards et al. 2008; Robu et al. 2013). However, such results should be considered with caution because they may be influenced by many other factors not related to diet, e.g. individual age, environmental conditions, climate, physiology and hibernation length (see Grandal-d’Anglade et al. (2011) and Pacher and Stuart (2009)), for discussion). Nevertheless, we cannot rule out some dietary flexibility of cave bears in their dependence on local food type accessibility. For example, *U. ingressus* from Loutra Arideas Cave in Greece was mainly herbivorous but probably fed also on aquatic animals, as it is transpires from isotopic studies (Dotsika et al. 2011).

Similarly, comparative analysis of dental microwear of specimens from Goyet Cave in Belgium did not support dietary specialisation of cave bears but rather indicated a mixed model based on plants, meat, invertebrates and supposedly bones (Peigné et al. 2009). However, these studies were criticised by Bocherens (2009) for choosing too specialised reference herbivorous bears and for missing comparability in extant bears with similar food preferences. Recent microwear studies associated with stable isotope analyses provided no evidence for the carnivory of the cave bear and revealed that this herbivorous bear showed some ecological flexibility, probably related to climate fluctuations and competition between various haplotypes (Münzel et al. 2014). Other microwear analyses led to a conclusion that some cave bears consumed more bones than the brown bear (Pinto Llona 2006, 2013). Together with tooth marks left probably by cave bears on bones (Pinto and Andrews 2004; Quilès et al. 2006; Rabal-Garces et al. 2012), the findings can also be interpreted as occasional changes in preferential herbivorous habits related to pre-hibernation period or seasonal resource availability. Supposedly, with the deteriorating high-quality plant material, mainly herbivorous cave bears were able to shift more to omnivorous diet. Given the great area inhabited by cave bears, it is not beyond the bounds of possibility that distinct populations developed adaptations to local environmental conditions.

Nevertheless, the available data seem to indicate that the cave bear was strongly dependent on plant food. Therefore, it seems reasonable to assume that its extinction was triggered off by a decrease in vegetation productivity resulting from climate cooling in the last glacial, especially in stadials GS-6, GS-5 and GS-3. In agreement with that, we found a significant correlation between Greenland ice core δ^18^O record being a good climate proxy and the distribution of the cave bear record described by the density function (averaged for Poz-61719 and GdA-3894). The calculated number of cave bear records decreased with the decline of δ^18^O value. The correlation coefficient was 0.24 (number of data = 464; *p* value = 3 × 10^−7^) in the long time span from 34,510 (the maximum of the distribution) to 25,250 cal. years BP (the mean date of Stajnia sample). In the short time period from 27,850 (the GI-3) to 25,250 cal. years BP, the correlation coefficient was 0.48 (*n* = 131; *p* value = 7 × 10^−9^). Our results correspond with the findings by Stiller et al. (2010), who also showed a stepwise decline of the cave bear population over 25,000 years. The extinction of more than ten other species was dated to the similar period (Cooper et al. 2015), pointing to the climate change as the global factor for the megafauna transformation. In contrast the to the cave bear, its close relative, the brown bear (*U. arctos*), did not experience a decline in its population size at that time (Stiller et al. 2010), which may result from its larger dietary flexibility as an omnivore. Simultaneously, the cave bear from France, Germany and Poland remained faithful to its vegetarian diet for a long period of time, despite climatic fluctuations as it is suggested by stable isotope studies (Bocherens et al. 2014; Krajcarz et al. 2014; Münzel et al. 2014). After the cave bear extinction, *U. arctos* took over its ecological and nutritional niche and more often used caves during dormancy (Bocherens 2015; Kurtén 1976; Münzel et al. 2011).

Our results show that the extinction of the cave bear falls within the middle of GS-3 stadial, one of the coldest phases of the last glacial period. The colder climate not only decreased availability of plant food but also extended the already long hibernation period of the cave bear (Rabeder et al. 2000). At that time, the bear was more susceptible to human hunting (Grayson and Delpech 2003; Kurten 1958; Münzel et al. 2011; Stiller et al. 2010) and attacks by predators, such as lions, hyenas and wolves (Diedrich 2014) for meat acquisition as well as competition for the shelter and denning space. It could be an important factor in its extinction, since the cave bear was more dependent on caves for hibernation than the brown bear or other species (Kurtén 1976; Rabeder et al. 2000). Evidence of hunting and exploitation of cave bears by Middle and Upper Palaeolithic humans was reported in many regions of Europe (Austria, Belgium, Czech Republic, Germany, Italy, Poland, Slovakia and Slovenia) (see Wojtal et al. (2015) for review). One of the spectacular examples is a flint projectile embedded in a vertebrae found in Höhle Fels in Swabian Jura (Germany), where human hunters contributed to the extinction of cave bears through a long period (Münzel and Conard 2004a, b; Münzel et al. 2011). Hofreiter et al. (2007) argued that there is no evidence for a significant climate change around the time of the cave bear’s replacement in Ach valley (Germany) and assumed that the most plausible explanation is an increasing pressure from humans and the difference in dietary habits of different haplotypes of the cave bear. Humans could also be responsible for the decrease in genetic diversity and the final extinction of the cave bear in Ardèche (France) (Bon et al. 2011).

### Spatial pattern of cave bear survival and extinction

The global picture of the cave bear genetic diversity shows that its populations may have declined from east to west (Stiller et al. 2014). It may be true but only on a continental scale (Asia–Europe), assuming that the youngest bears in Asia are represented by the samples from Ural dated to 35,773 and 41,026 cal. years BP (Pacher and Stuart 2009). They are much older than those from Europe. However, the amassed dates of the last records of the cave bear younger than 29,000 cal. years BP are distributed in distant sites in the whole of Europe, including not only western sites (Austria, Germany, France, Switzerland) and south (Croatia, Italy, Spain) but also eastern regions (Hungary, Poland, Slovakia)—Table 2. It seems to imply that the extinction of this mammal was preceded by a fragmentation of its populations into separated subpopulations, as it was found for bears from the north-western alpine foreland (Bocherens et al. 2014). It resulted from an ongoing increase of steppes, tundra and open landscapes at the expense of woodland, which were fragmented into smaller habitats. In such patchy woodland remnants with sufficient plant productivity, the cave bear could hold on longer (Martini et al. 2014).

One of such regions is likely to be the Kraków-Częstochowa Upland in Poland. This region is typically abundant in the cave bear remains. Besides Stajnia Cave, quite late cave bear remains were also found in other caves in this region, i.e. in Komarowa Cave (dated to 24,550 ± 220 ^14^C years BP), Deszczowa Cave (24,580 ± 200 ^14^C years BP) and Mamutowa Cave (26,010 ± 150 ^14^C years BP). It confirms that one of the latest cave bear populations could survive relatively long in this region. The numerous remains of the cave bear were found across the whole profile in the Stajnia Cave, from layers dating from more than 100,000 years (MIS 5c). It would be indicative of a region inhabited continuously by an abundant population of the cave bear till its extinction in the Greenland Stadial 3.

Despite the location of Kraków-Częstochowa Upland north from Carpathians, this region could be a refugial area because it was not covered by ice sheet during the last glacial. At the time of its maximum extent between ca. 23,000 and 19,500 years BP (Clark et al. 2009), Scandinavian Ice Sheet only reached the location of Płock and Konin, about 200 km north of the Stajnia Cave (Marks 2012). Furthermore, the central part of this upland was not glaciated either in the previous glacial periods owing to its elevation and unique configuration of the surrounding river valleys. As a result, ice sheets that came across this morphological step bypassed a “Glacial Oasis of the Polish *Jura* [‘Jurassic Highland’]” (Lewandowski 2011). Although a consistency between stratigraphy and radiocarbon age in cave sediments of the Kraków-Częstochowa Upland was not always found (Lorenc 2013), there are some results suggesting that this region could offer quite favourable environmental conditions for plants and animals even in stadials and play a role of local refugium for the forest fauna during the Saalian and Vistulian glaciations (Stefaniak et al. 2009). For example, the studies of Biśnik Cave located in the southern part of Kraków-Częstochowa Upland suggested that even in cold periods, birds and mammals typical for forest environments or associated with dense vegetation were present in the vicinity of the cave (Cyrek et al. 2010; Socha 2009; Stefaniak et al. 2009; Tomek et al. 2012). Detailed bioclimatic analyses based on the deposition of rodent assemblages in the cave’s layers from the late Middle and Late Pleistocene also indicated conditions of typical temperate climate in the biome of deciduous forests (Socha 2014). Interestingly, a temperature decrease deduced from the layers corresponding to the coldest stadials (e.g. the LGM) was smaller than it should be expected from global climatic conditions in these periods (Socha 2014). Similarly, the fauna assemblage found in the Borsuka Cave in the southern part of the Kraków-Częstochowa Upland also demonstrated the presence of forest adapted species at the end of the Upper Plenivistulian (Wilczyński et al. 2012). For example, in the layer dated to 32,000–31,000 cal. years BP, the elk *Alces alces* and the beaver *Castor fiber* were reported (Wilczyński et al. 2012). Assemblages dated to the LGM recovered from the Mamutowa Cave in the Upland are similar to other northern refugial faunas around the Carpathian Bow, whereas those from the Deszczowa contain forest-associated temperate or even thermophile species such as the pine marten *Martes martes* and the fat dormouse *Glis glis* (Nadachowski et al. 2009; Sommer and Nadachowski 2006). With the latter results, however, one should err on the side of caution, owing to the controversies over the stratigraphy and the age of the sediments in this cave (Lorenc 2013).

The Kraków-Częstochowa Upland is a typical karst region. It was suggested that such biotopes with suitable microclimate were pleasant for flora and fauna also in the context of the other late cave bear found in Chiostraccio Cave in Italy (Martini et al. 2014). In fact, karst regions are characterized by a diversified relief rich in slopes, ridges, valleys and sinkholes (dolines). It results in various amounts of solar radiation, shading and albedo on different parts of the slopes and in consequence air temperature, air humidity and soil moisture (Bárány-Kevei 1999; Bátori et al. 2014b; Bokwa et al. 2008). Lower parts of valleys and sinkholes offer colder and wet conditions but more stable temperature, whereas steep slopes (especially facing the south) are characterized by higher temperatures. Moreover, slopes with different geographic exposure obtain various amounts of radiation depending on the time of the day (Bárány-Kevei 1999; Bokwa et al. 2008). These local environment conditions strongly differentiate the composition of vegetation in karst regions on a local scale (Bátori et al. 2011, 2014a, b; Bokwa et al. 2008; Ozkan et al. 2010). Thanks to that, they could play a refugial role in the preservation of vascular plants providing primary habitats to other species (Bátori et al. 2011; Bátori et al. 2012; Ozkan et al. 2010), including herbivorous cave bear. The expositional and environmental differences in karst terrains could cause snow cover to melt in various periods and vegetation to be available to cave bears in more abundance for longer time in the year, especially on the south-facing slopes receiving more solar radiation and showing lower probability of ground frosts. It made the ecosystem less dependent on the global climate. Such local climatic diversity was described also for the Kraków-Częstochowa Upland with its consequence on vegetation composition and distribution (Bokwa et al. 2008; Medwecka-Kornaś and Kornaś 1977). Together with the favourable microclimate, the karst regions offered a permanent access to water supplied by deeper aquifers, whereas the high porous carbonate rocks were better protected against frost, which lead to greater stability of their slopes (Martini et al. 2014).

Our findings show that apart from the Mediterranean region with the assumed more temperate climate, there were also other areas with relatively favourable environmental conditions, enabling a prolonged survival of the vanishing species. This provides corroborative evidence for the concept of the cryptic northern refugia, which are areas at high latitudes characterized by a climate that allowed the survival of temperate species during the glacials (Schmitt and Varga 2012; Stewart and Lister 2001). Among several others, the Carpathian region is considered to be such refugial area, following the studies of plant and mammal fossils from the sites that proved the occurrence of temperate species during the LGM (Sommer and Nadachowski 2006; Stewart and Lister 2001). The idea of cryptic northern refugia was further supported by the genetic diversity of several modern species like the bank vole *Clethrionomys glareolus* (Kotlík et al. 2006; Wójcik et al. 2010), the common vole *Microtus arvalis* (Stojak et al. 2015) or the adder *Vipera berus* (Ursenbacher et al. 2006).

## Conclusions

The youngest record of the cave bear dated to ca. 26,000–25,000 cal. years BP and genetically confirmed as *U. ingressus*, together with its estimated extinction time, indicates that its isolated populations could survive into the middle of GS-3 stadial, the coldest phase of the Last Glacial. Its disappearance resulted from a climate cooling, which fragmented the habitats of the cave bear and reduced plant productivity that constituted its staple diet. Low temperatures also prolonged hibernation period for this bear, resulting in the animal becoming more vulnerable to attacks by predators and to human hunting. The cave bear was disappearing by separation into isolated populations confined to small habitats. One of them was karst terrains, e.g. the Kraków-Częstochowa Upland in Poland, where the latest so far record of the cave bear was discovered. The regions offered relatively favourable environmental conditions, with milder microclimate retaining sufficient ecosystem productivity. Thanks to that, cave bears could survive in these refugial areas longer. It cannot be ruled out that it survived the Last Glacial Maximum, as some of the specimens seem to indicate, but the data should be re-dated and confirmed by ancient DNA analyses. Further research is needed to fully understand paleoecology of this cave bear population.
